# Mitochondrial dysfunction in Parkinson’s disease – a key disease hallmark with therapeutic potential

**DOI:** 10.1186/s13024-023-00676-7

**Published:** 2023-11-11

**Authors:** Martin T. Henrich, Wolfgang H. Oertel, D. James Surmeier, Fanni F. Geibl

**Affiliations:** 1 Department of Psychiatry and Psychotherapy, Philipps University Marburg, 35039 Marburg, Germany; 2 Department of Neurology, Philipps University Marburg, 35043 Marburg, Germany; 3 Department of Neuroscience, Feinberg School of Medicine, Northwestern University, Chicago, IL 60611 USA

**Keywords:** Parkinson’s disease, Synuclein, Mitochondria, Mitochondrial dysfunction, MPTP, Electron transport chain, Antioxidants, Neuroprotective therapies

## Abstract

Mitochondrial dysfunction is strongly implicated in the etiology of idiopathic and genetic Parkinson’s disease (PD). However, strategies aimed at ameliorating mitochondrial dysfunction, including antioxidants, antidiabetic drugs, and iron chelators, have failed in disease-modification clinical trials. In this review, we summarize the cellular determinants of mitochondrial dysfunction, including impairment of electron transport chain complex 1, increased oxidative stress, disturbed mitochondrial quality control mechanisms, and cellular bioenergetic deficiency. In addition, we outline mitochondrial pathways to neurodegeneration in the current context of PD pathogenesis, and review past and current treatment strategies in an attempt to better understand why translational efforts thus far have been unsuccessful.

## Background

Parkinson’s disease (PD) is the most prevalent neurodegenerative movement disorder affecting up to 2 % of those aged 60 years and older [1]. Clinically, PD is defined by presence of the levodopa-responsive motor symptoms bradykinesia with resting tremor or rigidity [2]. These motor symptoms are frequently accompanied by non-motor symptoms, including but not limited to sleep disturbances, depression, autonomic dysfunction, and hyposmia [3, 4]. Today, there are still no proven strategies for slowing the progression of PD. This unmet medical need reflects our incomplete grasp of disease mechanisms.

Neuropathologically, PD is characterized by two imperfectly aligned features: selective neuronal degeneration of vulnerable cell-types within particular brain regions (e.g., midbrain dopaminergic (DA) substantia nigra pars compacta (SNc) neurons [5, 6]), and the presence of eosinophilic alpha-synuclein (aSYN) positive inclusion bodies, termed Lewy pathology (LP). Systematic cross-sectional characterization of human postmortem PD brains revealed that even in late-stage disease LP is not globally distributed in the brain of PD patients, but is restricted to certain vulnerable nuclei, thereby showing a patch-like distribution [7, 8]. While there is clear evidence that some regions (SNc, olfactory bulb, dorsal motor nucleus of vagus, locus coeruleus, pedunculopontine nucleus, amygdala) are more susceptible to LP than others, it has been difficult to establish the sequence and extent in which they develop LP. In addition to brain pathology, LP also affects many structures of the peripheral nervous system (nerve fibers within e.g., skin, heart, esophagus) [7, 9]. The observation that misfolded, fibrillar forms of aSYN can propagate from one cell to another in PD animal models [10], has fueled the thought that also in humans toxic aSYN species might spread between synaptically coupled brain regions, thereby driving the development of brain-wide LP formation [11].

In contrast to the relatively well-mapped distribution of LP, the spatio-temporal development of cell-loss within affected regions remains largely elusive. While loss of dopaminergic SNc neurons has been well-documented and clearly linked to the onset of PD motor symptomatology, there is no brain-wide assessment of neurodegeneration, and the available studies investigating cell loss show notable heterogeneity [12]. Given the absence of a clear correlation between LP formation and neuronal cell loss, it is crucial to disentangle the cell-intrinsic factors which render neurons susceptible to LP formation and those who drive neurodegeneration. So far, several core pathogenetic factors have been identified. Among those are impaired cellular protein homeostasis, dysfunctional proteasomal and lysosomal clearance systems, impaired protein and membrane trafficking, synaptic dysfunction including disturbed neurotransmission, neuroinflammation, and mitochondrial dysfunction [3, 13–16].

Mitochondrial dysfunction has long been implicated as a key pathological hallmark in PD. Since mitochondria are highly multifunctional organelles, their integrity is essential for neuronal function and survival. This review summarizes the evidence for mitochondrial dysfunction in genetic and idiopathic PD, discusses the bidirectional interaction between mitochondrial stress and aSYN aggregation, and points out potential mitochondrial pathways to neurodegeneration in the current context of PD pathogenesis. Further, we review current and past therapeutic strategies targeting mitochondrial dysfunction in an attempt to modify disease progression, and outline current gaps in our understanding.

## Main text

### Importance of mitochondrial health in PD at-risk neurons

Neurons possess a complex network of mitochondria stretching from dendrites that receive synaptic contacts to the synaptic terminals that communicate with neighboring neurons. Mitochondria perform a variety of tasks, including generation of adenosine triphosphate (ATP), Ca^2+^ buffering and epigenetic signaling [17–19]. Two central tenets of the mitochondrial theory of pathogenesis are that neurons have a high bioenergetic demand and that neurons rely heavily on mitochondria for ATP production. Indeed, all cells rely upon ATP to drive basic cellular processes. Neurons differ from many other cell types in ways that increase their bioenergetic needs. In particular, they need ATP to maintain ionic homeostasis which is being constantly challenged by 1) their reliance upon electrical signals generated by transmembrane ion fluxes, 2) their sequestration of transmitter into vesicles, fusion of these vesicles during synaptic activity and reuptake of membrane during vesicular recycling, and 3) the need to maintain and repair an often massive transmitter release machinery [20]. The ATP necessary for these processes can be derived both from glycolysis and mitochondrial oxidative phosphorylation (OXPHOS). While glycolytic mechanisms are fast, they are relatively inefficient and generate roughly one tenth the ATP from glucose that mitochondria can extract. It has been hypothesized that neurons rely exclusively upon mitochondrial OXPHOS for ATP generation (using lactate shuttled from astrocytes), but more recent direct measurements have shown that neurons use both glycolysis and OXPHOS to generate ATP [21].

Despite the clear importance of mitochondria to neuronal bioenergetics, they also play a variety of other roles. One of these is Ca^2+^ buffering. This may be particularly important in axons of some neurons [22]. Another important function is metabolic signaling [19]. For example, mitochondria are critical sources of citrate, which is important to the production of acetyl-coenzyme A and acetylation of proteins and DNA.

Compromised mitochondrial function may have a disproportionate impact on those neurons that are at-risk in PD. The best studied example of this phenotype is the SNc dopaminergic neuron. These neurons are constantly active and have extensive axonal arbors with as many as 1–2 million transmitter release sites per axon in humans [23]. Many (if not all) of the other neurons at greatest risk in PD have a similar phenotype: locus coeruleus noradrenergic neurons, dorsal motor nucleus of the vagus cholinergic neurons, and pedunculopontine nucleus cholinergic neurons [20, 24–26]. These neurons play a key role in organismal survival, particularly during times of crisis when sustained, efficient function is critical.

To meet this bioenergetic demand, many at-risk neurons engage a feed-forward control mechanism that utilizes plasma membrane L-type Ca^2+^ channels to drive mitochondrial OXPHOS [27–32]. While this feed-forward control helps to ensure that ATP levels do not fall during times of high demand, it also increases the production of damaging reactive oxygen species (ROS) and basal mitochondrial oxidant stress. ROS and mitochondrial oxidant stress damages lipids, proteins and DNA [33]. This can not only compromise cellular function but leads to an increased demand on catabolic processes in neurons, most importantly lysosomal degradation. This increased demand should in principle decrease spare capacity, providing a linkage between mitochondrial stress and genetic mutations linked to familial cases of PD involving mitochondrial quality control (DJ1, PINK1, parkin) and lysosomal function (GBA1, LRRK2, VPS35, others).

### Evidence for mitochondrial impairment in PD patients

**Fig. 1:**
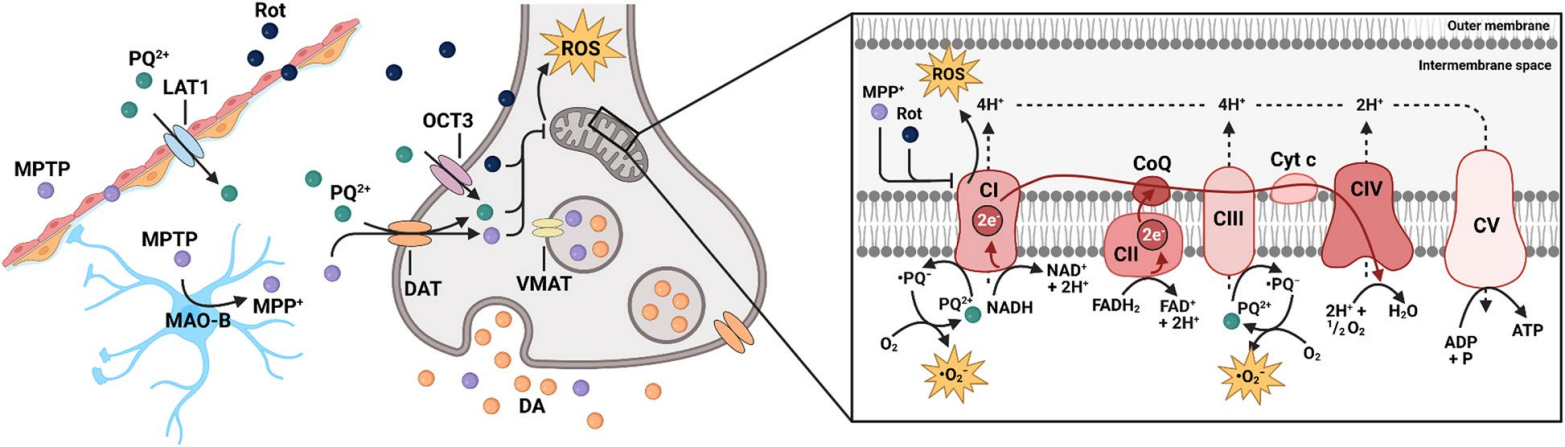
Mechanism of action of neurotoxins inducing PD. MPTP readily crosses the blood-brain barrier and is taken up by nearby astroglia which subsequently convert it to MPP^+^ via MAO-B. Extracellularly released MPP^+^ is then actively taken up via DAT and accumulates within mitochondria of DA neurons where it inhibits mitochondrial CI of the ETC resulting in ROS production and energetic deficiency. Similarly, the pesticide rotenone (Rot), due to its high lipophilicity, readily crosses biological membranes and reaches the inner mitochondrial membrane where it inhibits CI. In contrast, paraquat (PQ^2+^) relies on the LAT1 to cross the blood-brain barrier. Hereafter, it is taken up by DAT or OCT3 into DA neurons and generates ROS by redox cycling at CI and CIII of the ETC. Abbreviations: 1-methyl-4-phenyl-1,2,3,6-tetrahydropyridine (MPTP); 1-methyl-4-phenylpyridinium (MPP^+^); coenzyme Q (CoQ); dopamine (DA); dopamine transporter (DAT); L-amino acid transporter (LAT1); mitochondrial Complex I (CI); mitochondrial Complex II (CII); mitochondrial Complex III (CIII); mitochondrial Complex IV (CIV); mitochondrial Complex V (CV); monoamino oxidase B (MAO-B); organic cation transporter 3 (OCT3); paraquat (PQ^2+^); reactive oxygen species (ROS); rotenone (Rot); vesicular monoamino transporter (VMAT). Created with BioRender.com

A key piece of evidence that mitochondrial dysfunction is implicated in PD pathogenesis stems from the observation in 1983 that several recreational drug users which intravenously administered the new synthetic heroin drug MPPP (1-methyl-4-phenyl-4-propionoxy-piperidine) developed acute-onset but levodopa (L-DOPA) responsive parkinsonian motor symptoms shortly after drug administration [34]. Subsequently, the mitochondrial ETC inhibitor MPTP (1-methyl-4-phenyl-1,2,3,6-tetrahydropyridine) was identified as a byproduct of poor MPPP synthesis. Following absorption, MPTP crosses the blood-brain barrier and is converted to MPP^+^ within astroglia by monoaminoxidase B (Fig. 1). Extracellularly released MPP^+^ is then actively taken up via the DA transporter and accumulates within mitochondria of DA neurons where it inhibits mitochondrial complex I (CI) of the ETC [35–37]. Since its first discovery, MPTP induced toxicity has been established and validated many times as a reliable approach to model neurodegeneration and development of motor symptoms in rodents and primates [38, 39]. From a translational standpoint, the MPTP studies have taught us that mitochondrial CI inhibition in DA SNc neurons can cause a disease phenotype that resembles many features of idiopathic PD, e.g. all cardinal motor symptoms (bradykinesia, rigidity, tremor), some non-motor symptoms (dribbling of saliva, urinary disturbances), and L-DOPA responsiveness.

The observation that CI blockade can induce PD-like symptoms is further substantiated by the finding that the chemically related substance paraquat, as well as the CI inhibitor rotenone (Fig. 1), are not only shown by epidemiology to be risk factors for the development of PD, but also induce PD-like symptomatology in animal experiments [40–42]. More recently, genetic approaches have shown that disruption of CI function specifically in dopaminergic neurons is sufficient to produce a progressive, L-DOPA-responsive parkinsonism [43].

But is mitochondrial dysfunction also a constant and reliable feature of idiopathic PD, meaning in the absence of mitochondrial toxins or genetic disease drivers? Important aspects can be derived from the analyses of brain tissue from deceased idiopathic PD patients. In several studies, tissue samples of the SNc but also of other brain regions, as well as lymphocytes and platelets were analyzed for the presence of ETC alterations by immunoblotting, immunohistochemistry, or enzyme activity analysis. The most pronounced and consistently reported finding is a decreased activity of CI of the ETC in SNc tissue homogenates [44–47]. Some studies even observed a decreased activity of CI in tissue samples from the frontal as well as prefrontal cortices and striatum, but not peripheral tissues [48–50]. In contrast, deficiency of ETC CII, CIII or CIV was only sporadically observed, and when ETC function was either assessed by immunohistochemistry or other peripheral specimens (e.g. lymphocytes, platelets, or muscle) were analyzed, CI dysfunction was only inconsistently reported [47].

Regarding the lack of concordance in some studies it is important to consider that most experiments either used mixed tissue homogenates (neuronal and non-neuronal cells), or investigated peripheral tissue, which from our current point of view is likely not the main manifestation place of PD pathology. Future studies investigating ETC dysfunction in human postmortem tissue using now available cell-type specific approaches might therefore possess great potential to further enhance our understanding of mitochondrial dysfunction in idiopathic PD [43].

Another line of evidence pointing to mitochondrial dysfunction in PD is based on the observation of increased mitochondrial DNA (mtDNA) aberrations in tissue samples of deceased patients with idiopathic PD. While initial approaches investigating mtDNA deletions produced conflicting results [51–53], more recent studies confirmed an increased amount of mtDNA deletions specifically in postmortem SNc tissue of PD patients [54–56]. In addition, patients carrying a mutation of the polymerase gamma gene, the only polymerase present in human mitochondria, develop rare genetic syndromes including parkinsonian symptoms and loss of SNc neurons [57]. Taken together, there is mounting clear evidence implicating mitochondrial dysfunction as a key disease hallmark in idiopathic PD.

### Mitochondrial dysfunction is tightly linked to genetic PD

Although only roughly 10% of PD cases are associated with defined genetic alterations, the study of these familial PD (PARK) genes has led to major advances in our understanding of PD etiopathogenesis. While numerous PARK genes have been identified, several of these are directly linked to impaired mitochondrial function and integrity (Table 1).

**Table 1:** Mitochondria relevant PARK genes

Locus	Gene product	Inheritance	Progression	Physiological function	Pathological effect on mitochondria	Phenotype
PARK1/4	aSYN	Autosomal dominant	Rapid	Synaptic vesicle release/transmission [58]	Reduced complex 1 function, elevated mitochondrial ROS, impaired ATP-synthase function [59–66].	Age of onset: 30–50 years; Lewy pathology in humans: yes
PARK2	E3-Ubiquitin-protein-Ligase Parkin	Autosomal recessive	Slow	Mitochondrial quality control (mitophagy, fusion and fission, mitochondrial quality control) [67, 68]	Impaired mitophagy, impaired mitochondrial biogenesis, defects of mitochondrial structure [67, 69].	Age of onset: approx. 30 years; Lewy pathology in humans: variable
PARK6	PTEN-induced kinase-1 (PINK1)	Autosomal recessive	Variable	Mitochondrial quality control (mitophagy, fusion and fission, mitochondrial derived vesicles) [67, 70].	Impaired mitophagy, impaired mitochondrial biogenesis, defects of mitochondrial structure, ETC impairment and reduced ATP production, high levels of ROS [71–73].	Age of onset: 30–50 years; Lewy pathology in humans: variable
PARK7	Protein deglycase DJ1 (DJ1)	Autosomal recessive	Slow	Counteracting oxidative stress. Additional chaperone activity. Role in ER-mitochondrial calcium homeostasis [74–76].	Elevated levels of ROS, decreased mitochondrial membrane potential, altered mitochondrial morphology [77–80].	Age of onset: 20–40 years; Lewy pathology in humans: yes
PARK8	Leucine-rich repeat kinase 2 (LRRK2)	Autosomal dominant	Fast	Protein with two enzymatic activities (kinase and GTPase) involved in a plethora of cellular signaling [81, 82].	Indirect effect on mitochondria via modulation of lysosomal degradation and cytoskeleton. Also, direct effect causing impaired mitophagy, altered fusion and fission, impaired trafficking and increased ROS [83–86].	Age of onset: Typically, 50–60 years, albeit early (< 30s) and late (> 80s) onset has been reported [87, 88]. Lewy pathology in humans: variable
PARK9	ATPase type 13A2 (ATP13A2)	Autosomal recessive	Slow	Primary involvement in lysosomal system [89, 90].	Increased ROS and mitochondrial fragmentation, increased aSYN aggregation [91–93].	Age of onset: 10–20 years; Lewy pathology in humans: unknown
PARK15	F-box protein 7 (FBXO7)	Autosomal recessive	Rapid	Neuronal role still largely unclear. Might interact with Parkin and promote mitophagy [94, 95].	Impaired mitophagy, decreased complex 1 function [96, 97].	Age of onset: 10–20 years; Lewy pathology in humans: unknown
PARK17	Vacuolar protein sorting 35 homolog (VPS35)	Autosomal dominant	Slow	Part of the cellular retromer complex and relevant for intracellular trafficking. Implicated in generation of mitochondrial derived vesicles and lysosomal degradation [98, 99].	Increased mitochondrial fission and fragmentation, complex 1 impairment [100–103].	Age of onset: approx. 50 years; Lewy pathology in humans: unknown
PARK22	Coiled-coil-helix-coiled-coil-helix domain containing protein 2 (CHCHD2)	Autosomal dominant	Slow	Mitochondrial protein. Stabilizing effect on cristae structure. Regulating mitochondrial stress response [104, 105].	Abnormal mitochondrial structure, impaired mitochondrial respiration, elevated ROS levels, aggregation of aSYN [106–108].	Age of onset: 50 years; Lewy pathology in humans: yes
PARK23	Vacuolar protein sorting 13 homolog C (VPS13C)	Autosomal recessive	Rapid	Lipid transport protein implicated in mitochondrial biogenesis and mitophagy [109, 110].	Abnormal mitochondrial morphology, disturbed mitochondrial membrane potential, increased mitophagy [100].	Age of onset: 20–30 years; Lewy pathology in humans: yes

Mutations of the genes coding for PINK1 (PARK6) and Parkin (PARK2) are the most frequent causes of autosomal recessive early-onset PD. Their clinical manifestation is characterized by relatively pure motor symptomatology and L-DOPA responsiveness, which can be accompanied by dopamimetica associated dyskinesia, hyperreflexia, and sometimes psychiatric symptoms. Interestingly, histopathological examination of postmortem tissue indicates loss of SNc dopaminergic neurons and neurons in other brain regions normally vulnerable in idiopathic PD (e.g., locus coeruleus, nucleus basalis meynert). However, presence of aSYN inclusions, a hallmark of idiopathic PD, is not a consistent feature of these PD cases [111–116]. At the cellular level, PINK1 and Parkin play key roles in mitochondrial quality control mechanisms and signaling cascades in response to mitochondrial damage [67]. PINK1/Parkin can not only initiate mitophagy, but also control fission and fusion of mitochondria, promote the generation of mitochondria derived vesicles and induce mitochondrial biogenesis [70, 117–121]. In fibroblasts from PINK1 and Parkin familial PD cases, loss of protein function leads to ETC impairment with reduced ATP production and high levels of ROS [122–124]. While experimental studies using Parkin-KO mice revealed lower levels of mitochondrial respiratory capacity [125–127], PINK1-KO mice additionally exhibited defects in CI function, reduced Ca^2+^ buffering capacity, and impairments in mitochondrial membrane potential [128–131]. Comparable findings have also been reported in Drosophila Parkin and PINK1 models [71–73, 132–134]. As the underlying pathophysiological event, increased mitochondrial fission has been identified in Parkin and PINK1 mutant mice and Drosophila models [117, 135]. This is supported by the fact that inhibition of mitochondrial fission via mdivi-1 treatment, was able to rescue mitochondrial function by normalizing the balance between mitochondrial fission and fusion [136]. Apart from increased fission, defects in mitochondrial biogenesis have been shown to contribute to mitochondrial dysfunction in Parkin deficient human dopaminergic neurons [121].

Interestingly, there is additional evidence for accumulation of insoluble Parkin within idiopathic PD patients. While previous studies observed that accumulating Parkin is S-nitrosylated [137–140], a more recent study discovered that Parkin itself functions as a redox molecule by providing antioxidant capacity for human midbrain neurons. Subsequent oxidizing posttranslational modifications then contribute to the decrease in Parkin solubility [141].

Another example indicating mitochondrial driven parkinsonism, are mutations in the gene coding for DJ1 (PARK7). Resulting loss of function leads to an autosomal recessive form of PD which is less common than PINK1 or Parkin familial PD. The clinical presentation of individuals with DJ1 mutations is characterized by early onset slow progressing parkinsonism, which is frequently accompanied by non-motor symptomatology (e.g., anxiety, cognitive decline, and psychotic symptoms), and good L-DOPA responsiveness [142]. Notably, postmortem histopathological analysis revealed widespread cortical and subcortical LP and neurodegeneration [77]. DJ1 is involved in counteracting oxidative stress and subsequent mitochondrial dysfunction under physiological conditions. In the experimental setting, DJ1 depletion leads to impaired mitochondrial respiration, high levels of intracellular ROS, compromised mitochondrial membrane potential, and altered mitochondrial morphology [78, 143–145]. Furthermore, mutated DJ1 is translocated from the cytosol into the mitochondrial matrix where it gets degraded [146]. Despite the increasing interest in DJ1’s function, the molecular mechanisms remain incompletely understood. Several lines of evidence suggest that DJ1 is a redox-sensitive protein which relies on cysteine oxidation to sense oxidative stress and then counteract this stress through activation of different signaling pathways [147–149]. Other reports suggest that DJ1 may additionally possess chaperone activity [150, 151], supported by data showing that DJ1 is able to attenuate aSYN aggregation [152], and the observation that human induced pluripotent stem cells (IPSCs) derived from fibroblasts of DJ1 PD patients exhibit increased aSYN pathology [153]. However, further evidence highlights DJ1’s enzymatic functions, including glyoxalase and deglycase activities, showing that DJ1 can decrease reactive carbonyl products and repair glycated nucleic acids [154, 155]. Albeit the exact biological interplay of these processes is still debated, DJ1 clearly links antioxidant pathways, mitochondrial dysfunction, and aSYN aggregation.

More recently mutations affecting vacuolar protein sorting 35 (VPS35 = PARK17) have been linked to late-onset autosomal dominant PD, and VPS13C (PARK23) to early onset rapid progressing autosomal recessive PD [100, 156, 157]. Although the exact pathophysiological mechanisms are still intensively debated, experimental studies on VPS35 mutant fibroblasts, mice, or cell culture systems reported increased mitochondrial fragmentation, disturbed mitochondrial fission and fusion dynamics, and abnormal configuration of ETC CI [101–103, 158]. Mechanistically, VPS35 is a part of the retromer complex and thereby plays an important role in endosomal sorting and trafficking of proteins. VPS35 mutations have been shown to lead to an enhanced interaction of VPS35 with DLP1, which subsequently causes increased turnover of mitochondrial DLP1 complex, thereby fueling excessive mitochondrial fission, finally culminating in mitochondrial dysfunction and fragmentation [101, 158]. Further, VPS13C mutations have been shown to decrease mitochondrial membrane potential, promote mitochondrial fragmentation, and elevate mitophagy [100]. In addition, mutations in FBXO7 (PARK15), causing a rare syndrome of juvenile parkinsonism with pyramidal signs, have been linked to impaired mitophagy and decreased CI function [94].

Taken together, the familial PD cases not only show us that there is a clear link between genetic PD and mitochondrial dysfunction, they also highlight that multiple mitochondrial pathways may be impaired, including CI function, mitophagy, fission and fusion, and mitochondrial biogenesis.

### Causal link between α-synuclein pathology and mitochondrial dysfunction

**Fig. 2:**
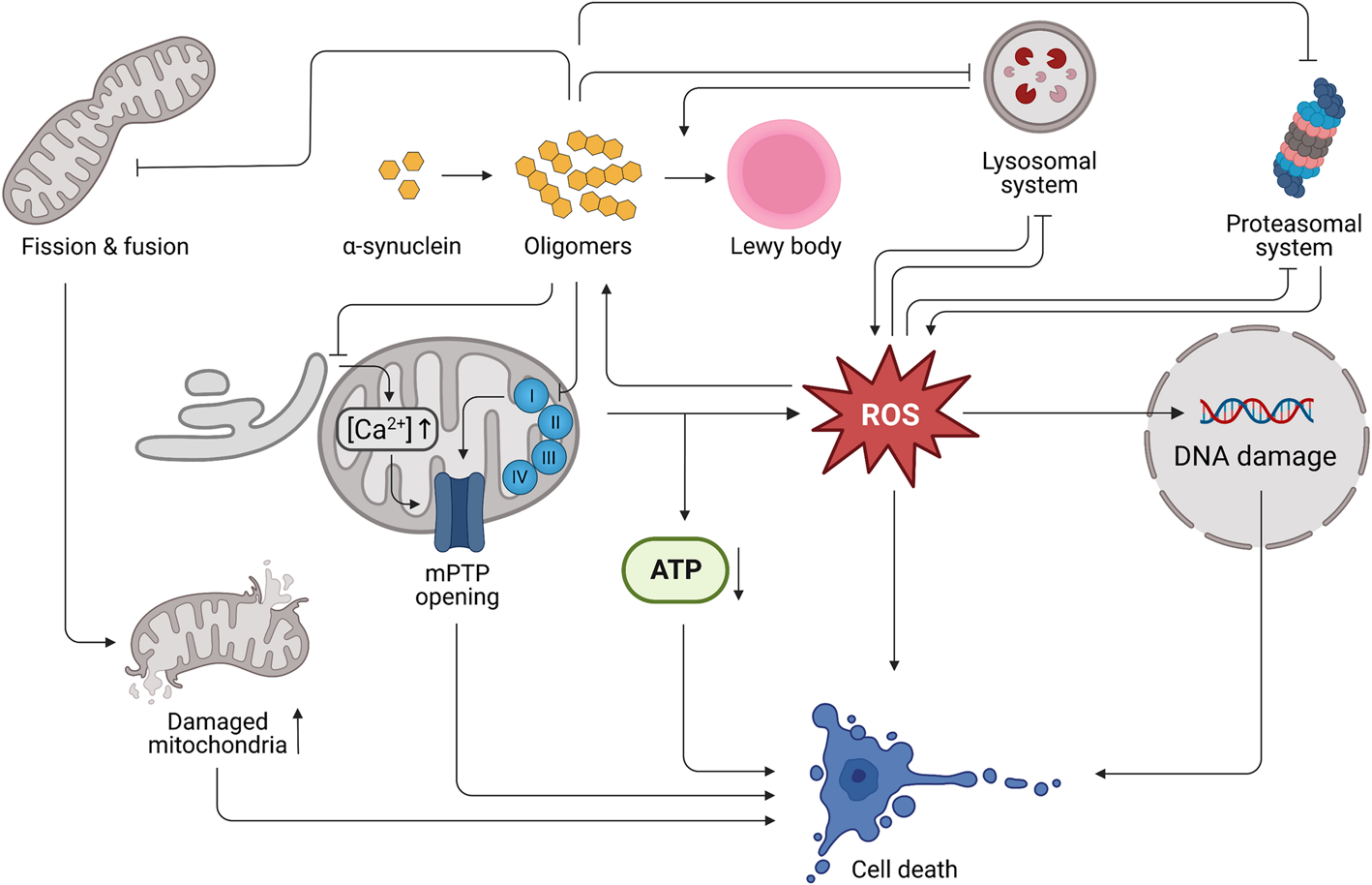
Synucleinopathy-driven mechanisms of cellular dysfunction and death in PD. Abbreviations: mitochondrial permeability transition pore (mPTP); reactive oxygen species (ROS). Created with BioRender.com

While for a portion of PD patients, the occurrence of mitochondrial dysfunction can be explained by PARK genes, the etiology of idiopathic PD is still a matter of intensive debate. However, broad experimental evidence stemming from observations in isolated mitochondria [59–61, 159], and rodents [62, 63], suggests aSYN pathology as a major source of mitochondrial dysfunction (Fig. 2). Under physiological conditions, monomeric aSYN was shown to modulate the function of the mitochondrial ATP synthase subunit alpha, as aSYN knock-out mice showed reduced ATP synthase efficiency and reduced ATP levels [64]. Similarly, another study employing aSYN deficient mice observed an altered neuronal mitochondrial membrane structure and CI deficiency [65].

In the presence of aSYN pathology, meaning excessive amounts of overexpressed monomeric aSYN or existence of oligomeric and fibrillar aSYN, several studies reported decreased mitochondrial CI activity, alterations of mitochondrial membrane potential, and elevated oxidative stress levels [59, 63, 66, 160–162]. The effect on CI is further substantiated by another study which reported a dose-dependent effect of aSYN pathology on CI inhibition [163]. Based on the observation that aSYN knock-out mice were resistant to MPTP induced toxicity, it has been hypothesized that aSYN directly influences CI function [35, 164]. This view is supported by studies which reported that overexpression of human aSYN in wildtype mice or use of SNCA A30P mutated transgenic mice worsened MPTP induced toxicity [165, 166]. Similar findings have also been observed for the CI inhibitor rotenone [167, 168]. However, CI does not seem to be the only engagement point for aSYN pathology. More recently, interaction of pathological aSYN oligomers with the ATP synthase subunit alpha in combination with mitochondrial permeability transition pore opening has been suggested as a mediator of aSYN induced mitochondrial dysfunction [169]. Further, it has been shown that aSYN oligomers interact with the outer mitochondrial membrane protein TOM20 [170, 171]. As a consequence of aSYN binding to TOM20, mitochondrial protein import is impaired causing ETC malfunction, accumulation of ROS and loss of mitochondrial membrane potential [170]. aSYN induced loosening of contacts between mitochondria and the endoplasmic reticulum (ER), which are considered essential for proper Ca^2+^ exchange between those two organelles, has been reported as another possible cause of reduced mitochondrial respiration, primarily by dysregulated intracellular Ca^2+^ levels [172, 173].

Taken together, these studies not only show that aSYN pathology can trigger mitochondrial dysfunction, they reveal that there are several independent pathways how aSYN pathology affects mitochondrial function (Fig. 2). Notably, many of those pathways converge to a shared pathological phenotype exhibiting increased cellular and mitochondrial ROS, impairment of mitochondrial membrane potential, and reduced mitochondrial respiration.

### Pathways linking mitochondrial dysfunction to neurodegeneration

Does mitochondrial dysfunction cause neurodegeneration in PD, or is it simply a disease tombstone?

While this question is difficult to answer for idiopathic PD, important information can be gleaned again from familial PD cases by looking at those few histopathological postmortem reports which are available. Notably, PINK1 as well as Parkin, and DJ1 mutation carriers, all familial PD cases where PD is thought to be majorly driven by mitochondrial dysfunction, exhibit marked neuronal cell loss within the SNc and other susceptible brain regions [77, 111, 112]. This clearly indicates that at least genetically driven mitochondrial dysfunction is causative of neuronal cell loss in these individuals. This is supported by the finding that targeted disruption of mitochondrial CI in mice leads to dopaminergic degeneration culminating in a human-like type of parkinsonism [43]. However, what is less clear is whether mitochondrial dysfunction is necessary for PD.

As mentioned above, mitochondrial dysfunction and damage can contribute to several pathological cascades implicated in PD [67, 174, 175]. As shown by using direct ratiometric probes, many at-risk neurons have been found to manifest elevated levels of mitochondrial oxidant stress [30, 31, 176]. Sustained oxidant stress damages membranes, proteins, and DNA. This damage elevates mitophagy in SNc dopaminergic neurons [176], thereby diminishing the overall autophagic capacity. Cytosolic ROS can further damage proteins of the mitophagy pathway [138] and increase mitochondrial dysfunction. Mitochondrially-generated ROS also compromises lysosomal and proteasomal function and increases the accumulation of misfolded forms of aSYN [153, 177]. Further, intracellular ROS triggers induction of parthanatos, an apoptosis independent pathway of neurodegeneration [178]. In parallel, damaged mitochondria or excessive mitochondrial stress can induce mtDNA release into the cytosol and subsequent increases in the production of proinflammatory cytokines [179, 180], as shown in Parkin-KO mice which also exhibited a POLG mutation [181]. Mitochondrial dysfunction is further connected to neuroinflammation by the observation that loss of PINK1 and Parkin function results in increased mitochondrial antigen presentation and subsequent activation of cytotoxic T-cells [182]. Intestinal infection with Gram-negative bacteria in PINK1 mice enhanced mitochondrial antigen presentation which was followed by elevated levels of CD8^+^ T-cells in the brain and periphery [183].

As indicated above, failure of mitochondrial quality control mechanisms defines another pathway to neurodegeneration in PD. Substantial evidence shows that the concerted interplay of PINK1 and Parkin is essential for maintaining mitochondrial health. Loss of function mutations result in disruption of cellular mitophagy, as well as impaired fusion and fission of mitochondria, and reduced generation of mitochondrial derived vesicles [67]. As a consequence, damaged mitochondria accumulate, cytochrome c and other proapoptotic proteins are released into the cytosol, and apoptosis might be induced. Damaged mitochondria due to loss of mitochondrial quality control mechanisms also contribute to the generation of oxidative stress and mtDNA mutations. Importantly, in idiopathic PD, LP also directly inactivates Parkin and thereby contributes to failure of mitochondrial quality control even in the absence of genetic mutations [138, 139].

Intracellular Ca^2+^ signaling also may contribute to pathogenesis [5]. At-risk neurons have low intrinsic Ca^2+^ buffering capacity and strong engagement of both plasma membrane and ER-dependent Ca^2+^ signaling, leading to large cytosolic oscillations in intracellular Ca^2+^ concentration [176]. Elevated intracellular Ca^2+^ can promote aSYN misfolding and aggregation [184, 185] thereby linking aSYN and Ca^2+^ in a vicious cycle.

Another key hallmark of PD is impaired cellular proteasomal and lysosomal mechanisms [153, 186, 187]. Proteasomal degradation as well as lysosomal function are energy consuming processes. It is easy to infer that compromised ATP production by mitochondria will reduce their functional capacity. Thus, elevated mitochondrial ROS production – and the resulting cellular damage – not only increases the burden on these systems, but with declining mitochondrial capacity it will likely diminish their capacity. As a consequence, not only aSYN aggregation is promoted but clearance of oxidized proteins is reduced, leading to further generation of ROS and oxidative damage in terms of a feedforward mechanism. Moreover, there is evidence for dynamic mitochondria-lysosome contacts which allow inter-organelle crosstalk. Interestingly, patient derived neurons harboring a heterozygous mutation within the gene coding for β-glucocerebrosidase (*GBA1*) show disturbed loosening of these contact sites which resulted in prolonged tethering and disruption of intracellular mitochondrial distribution [188].

Taken together, current evidence indicates that there are several mitochondrial pathways which are tightly linked to other pathogenic mechanisms of PD. While some of these pathways are highly interdependent, others act in parallel to each other. From a translational standpoint, this suggests that, as in cancer, new therapeutic approaches will either need to target several of these pathways at once or be tailored to pathological endpoints shared by these pathways.

### Therapeutic approaches targeting mitochondrial dysfunction in PD

One of the greatest challenges facing the biomedical community is the development of a disease-modifying therapy for PD. Several clinical trials have been attempted to address this challenge, but none have succeeded. Several have targeted mitochondrial function either directly or indirectly.

Given the recognition that mitochondrial oxidant stress is a potential driver of pathogenesis, some of the earliest trials aimed at reducing it (Table 2). For example, the antioxidant coenzyme Q10 (CoQ10) was tested in several trials, as was minocycline; they all failed [252, 253]. Mitochondrially-targeted antioxidants, like MitoQ, MitoVitE, MitoApocynin and MitoTEMPOL were developed to achieve better target engagement and showed promise in pre-clinical experiments, but this general strategy has not shown a clear benefit in PD patients [207]. One of the key issues with these trials is that it is difficult to demonstrate adequate target engagement and biological efficacy of these compounds in humans. So, it is unclear whether they are testing the core hypothesis or not.

**Table 2:** Current therapeutic approaches targeting oxidative stress

Antioxidants	Scientific basis	Results from preclinical studies	Results from clinical trials
Coenzyme Q_10_ (CoQ_10_), and derivatives (EPI-589, MK-7)	– Numerous studies have shown decreased levels of CoQ_10_ in plasma, platelets and distinct brain regions of PD patients [189]. – Evidence points to the role of elevated oxidative stress in the pathophysiological process of PD [190, 191]. – CoQ_10_ is thought to be a potent antioxidant [192].	– CoQ_10_ reduced rotenone-induced apoptosis and mitochondrial depolarization of primary rat mesencephalic neurons [193]. – Intrastriatal administration of CoQ_10_ showed neuroprotective effects in a 6-OHDA rat model of PD [194]. Oral administration of CoQ_10_ showed similar effects in a rotenone-induced rat model [195] and an MPTP-induced mouse model of PD [196, 197]. – Observed cellular effects included decreased ROS levels, normalized mitochondrial membrane potential, restored ATP generation.	– CoQ_10_ phase I trials revealed good safety and tolerability profile. Possible clinical benefit in a phase II trial [198]. However, phase III trial revealed no disease-modification potential [199, 200]. – EPI-589 exhibited a good tolerability profile in a recent phase I trial [201]. MK-7 is currently tested in a placebo-controlled pilot study [202].
MitoQ, MitoVitE, MitoApocynin, MitoTEMPOL	– Mitochondria targeted antioxidant approaches. These modified compounds are suggested to show enhanced mitochondrial target engagement compared to parent antioxidant [203].	– MitoQ has been shown to exert neuroprotective effects against MPTP induced neurotoxicity in primary mesencephalic neuronal cells and cultured dopaminergic cells as well as in the MPTP mouse model of PD [204]. – MitoApocynin has been shown to reduce dopaminergic neurodegeneration and neuroinflammation as well as ameliorate mitochondrial function in a MitoPark transgenic mouse model [205]. It also prevented hyposmia and motor symptoms in a LRRK2 (R1441G) transgenic mouse model. It has also been shown to protect against MPTP-induced neurotoxicity in vitro and in vivo [206].	– MitoQ failed to slow PD progression in de novo PD patients in a double-blind placebo-controlled trial [207]. – Robust clinical data for other mitochondria targeted compounds are lacking.
GPI1485	– Belongs to the group of neuroimmunophilins and is supposed to exhibit neurotrophic and antioxidative effects [208].	– No study exists investigating the neuroprotective efficacy of GPI1485 in in vitro or in vivo models of PD.	– Clinical trial data is inconclusive regarding a disease modifying effect [209].
Glutathione	– Robust evidence for reduced levels of glutathione in PD patients exists [210]. – Oxidative stress and ROS production are key pathophysiological events in PD [190]. Glutathione is a major cellular antioxidant [211]. It reduces cellular ROS and helps maintaining healthy neuronal redox state.	– Glutathione provided neuroprotection against paraquat plus maneb induced toxicity in rat mesencephalic mixed neuronal/glial cultures [212].	– Intranasal application of glutathione showed no disease modifying effects in a phase IIb study in manifest PD patients [213].
N-Acetyl-cysteine (NAC)	– NAC elevates cellular glutathione levels, thereby promoting antioxidant effects.	– Intraperitoneally administered NAC significantly ameliorated rotenone-induced motor dysfunction and dopaminergic neuronal cell loss in rats [214]. It has also been shown to exert neuroprotective effects in a 6-OHDA rat model of PD [215]. – Orally administered NAC attenuated the loss of striatal dopaminergic terminals in transgenic, wild-type aSYN overexpressing mice [216].	– NAC administration over 3 months in PD patients resulted in improvements in PD symptoms and dopaminergic imaging (DaTScan) [217].
Nicotinamide adenine dinucleotide (NAD)	– Broad evidence for NAD deficiency in PD [218].	– Injection in the striatum ameliorated 6-OHDA-induced dopaminergic neurodegeneration and motor deficits in a mouse model of PD [219]. Additionally, it prevented 6-OHDA-induced neuronal cell loss in vitro. – Additional evidence for direct improvement of mitochondrial function in PD patient derived iPSCs [220].	– Oral NAD treatment has been deemed safe and was well-tolerated in several clinical phase I trials [221, 222]. – Recent phase I trial indicated elevated brain NAD levels under treatment going along with clinical improvement of de novo PD patients [223]. Additionally, it induces upregulation of mitochondrial, antioxidant and proteasomal genes [223].
Inosine	– Inosine represents a metabolic precursor of the naturally occurring antioxidant urate. – Elevated serum urate levels in healthy individuals are associated with reduced risk for developing PD [224].	– Urate exerted antioxidative effects in primary midbrain dopaminergic and MES 23.5 cell cultures leading to long-term protection [225–227]. – Elevated brain urate levels attenuated toxic effects of intrastriatal 6-OHDA injection in mice [226]. – Oral inosine exerted neuroprotective effects and ameliorated motor deficits and dopaminergic neurodegeneration in the rotenone and the MPTP mouse and rat model of PD [228–230].	– Inosine was able to elevate patient urate levels, and deemed safe in a phase I trial [231]. – However, a recent randomized, double-blind, placebo-controlled, phase III trial of oral inosine treatment in early PD revealed no effect on disease progression [232].
Ursodeoxycholic acid (UDCA)	– UDCA possesses among anti-apoptotic, and anti-inflammatory characteristics, also the ability to stabilize mitochondrial integrity.	– In the rotenone and MPTP mouse models, UDCA treatment rescued mitochondrial integrity, normalized mitochondrial membrane potential and lead to increased levels of ATP and decreased levels of ROS [233–236]. – UDCA treatment of primary human fibroblast cultures of LRRK2G2019S mutations carriers ameliorated mitochondrial function and ATP production [234].	– UDCA exhibits a well-characterized safety profile and was able to increase brain ATP levels of PD patients in a pilot study [237]. – A phase II, placebo-controlled trial is currently ongoing, testing UDCA’s potential to slow PD progression and the ability to increase brain ATP levels (NCT03840005).
Minocycline and creatine	– Minocycline and creatine likely own anti-apoptotic, anti-inflammatory, antioxidant, and bioenergetic effects [238]. – Oral supplementation of creatine increases longevity in mice [238].	– Minocycline protected dopaminergic neurons in MPTP and 6-OHDA toxin rodent models of PD [239]. – Creatine rescued dopaminergic nigral neurons from MPTP toxicity in a rodent model of PD [240].	– Within a phase II trial neither minocycline nor creatine was deemed futile, but larger trials are needed [241]. – However, placebo-controlled trial in multiple system atrophy patients did not show disease modifying potential of minocycline [242].
Deferiprone	– Deferiprone is a well-established iron chelator. – Intracellular iron is elevated in PD patients and linked to increased levels of oxidative stress, and chelation of iron might therefore possess neuroprotective potential [243, 244].	– Deferiprone attenuated 6-OHDA as well as MPTP induced dopaminergic neurodegeneration in rodent models of PD [245–247]. – Deferiprone ameliorated MPP^+^ induced cytotoxicity of SHSY-5Y cells [248].	– Initial clinical trials indicated that deferiprone was able to decrease brain iron content [249, 250]. – However, in a recent phase II trial (FAIRPARK-II), involving de novo PD patients, deferiprone treatment led to an increase in parkinsonian symptoms and was found unsuitable for PD treatment [251].

A related approach is to try and boost brain concentrations of glutathione (Table 2). Nigral levels of glutathione are lower in PD patients, possibly because of an increased reliance upon glycolysis for ATP production in PD patients [210]. Elevating glutathione has been proposed and explored in preclinical and clinical trials [213]. However, it is unclear whether this is simply an effect of mitochondrial dysfunction and whether adequate brain concentrations can be achieved with oral dosing. N-Acetyl cysteine (NAC), an approved drug to treat acetaminophen induced liver failure [254], increases cellular glutathione levels in vivo. Notably, weekly intravenous administration of NAC over 3 months in idiopathic PD patients revealed a significant clinical improvement which was paralleled by increased dopamine transporter binding during ioflupane imaging (DaTSCAN) [217].

Another consequence of mitochondrial dysfunction is a lowering of nicotinamide adenine dinucleotide (NAD) [255]. Mitochondrial CI metabolizes NADH to NAD+. Boosting cellular NAD levels by dietary supplements of the precursor nicotinamide (vitamin B3) has neuroprotective effects in some preclinical models of PD [220]. The recent phase I study NADPARK in which drug naïve de novo PD patients received 1000 mg of nicotinamide riboside over 30 days achieved some desired metabolic outcomes and a mild clinical benefit [223].

A related approach is based upon epidemiological studies showing reduced risk of developing PD when using antidiabetic drugs like exenatide or pioglitazone [256]. Both drugs have been studied intensively in preclinical animal models and clinical PD trials (Table 3). Exenatide appears to exert its neuroprotective effects by dampening neuroinflammatory pathways, reduction of ROS, lowering intracellular Ca^2+^ levels, restoring mitophagy, and improving overall bioenergetic efficiency [258]. In a randomized double-blind placebo-controlled trial on PD patients under symptomatic dopamine replacement therapy, 48 weeks of exenatide, slightly although significantly, improved motor symptoms [260]. Currently, a phase III trial [261] is investigating the effects of a two-year exenatide treatment on motor symptoms in PD patients, which are again also receiving symptomatic dopamine replacement therapy. In contrast, 52 weeks long treatment with liraglutide, also a glucagon-like peptide 1 (GLP-1) agonist, resulted in improvement of non-motor symptoms and activities of daily living while motor symptoms were unchanged [262]. While preclinical models suggest a mitochondria-based mechanism of action, there is no robust data from clinical studies regarding GLP-1 agonist’s cellular mechanism of action.

**Table 3:** Therapeutic approaches targeting metabolism or mitochondrial quality control

Substance	Scientific basis	Results from preclinical studies	Results from clinical trials
Metabolic remodeling			
Glucagon-like peptide (GLP-1) agonists, e.g. exenatide, liraglutide	– Antidiabetic therapy with GLP-1 agonists (e.g., exenatide, liraglutide) is associated with reduced risk for developing PD.	– Preclinical studies in toxin-induced rodent models of PD (MPTP, rotenone, and 6-OHDA) suggested a neuroprotective potential for GLP-1 agonist treatment [257]. – The mechanism of action is still unclear. Currently, modulation of neuroinflammatory pathways, reduction of ROS, normalization of cellular Ca^2+^ levels, restoring mitophagy and improving overall bioenergetic efficiency is suggested [258]. – However, conflicting results exist, indicating exenatide may worsen aSYN accumulation [259].	– In a placebo-controlled trial exenatide treatment over 48 weeks resulted in clinical improvement of motor symptoms [260]. – A phase III trial (Exenatide-PD3; NCT04232969) is currently investigating the effect of a two-year exenatide treatment on motor symptomatology in PD patients [261]. – In a small double-blind, placebo-controlled, trial 52 weeks of liraglutide treatment resulted in significant improvement of non-motor symptoms and activities of daily living, while severity of motor symptoms was unchanged [262].
Peroxisome proliferator- activated receptor- γ (PPARγ) agonists, e.g., pioglitazone	– Antidiabetic treatment with pioglitazone is associated with a reduced risk for developing PD [256].	– Pioglitazone showed neuroprotective potential in a transgenic mitochondrial complex IV deficient mouse line of PD. – It also attenuated MPTP-induced dopaminergic neurodegeneration in a rodent model of PD [263]. – Mechanism of neuroprotection is unclear. Modulation of different cellular pathways including reduced neuroinflammation, suppressed nitric oxide synthase activity, improved proteasomal clearance, and enhanced mitochondrial biogenesis have been suggested [264].	– A phase II clinical trial investigating pioglitazone treatment over 44 weeks revealed no modification of disease progression in early PD patients [265].
Enhancing mitochondrial quality control			
mdivi-1	– Excessive Drp1-mediated mitochondrial fission has been identified as a pathomechanistic pathway in PD [266]. – mdivi-1 blocks Drp1.	– mdivi-1 reduced proteinase K resistant aggregates and mitochondrial ROS production as well as improved autophagy and ATP production in aSYN overexpressing or PFF exposed cells in vitro [267–269]. – mdivi-1 rescued the motor phenotype und exerted neuroprotective effects in A53T-aSYN overexpressing rats, the rotenone- or MPTP-induced rodent model of PD, [267, 269] as well as in the PINK-KO [270] mice.	– mdivi-1 has not been tested in clinical trials yet.
Miro-targeting	– Prolonged retention of the outer mitochondrial membrane protein Miro on mitochondria disturbs mitophagy and thereby contributes to PD pathology [271].	– Reduction of Miro rescued mitophagy in human fibroblast cultures of PD patients and Drosophila models of PD [272, 273].	– Miro reducers have not been tested in clinical trials.
Increasing PINK1/Parkin levels	– Deficits in PINK1 and/or Parkin signaling are known causes of genetic PD.	– Several preclinical studies indicated that increasing levels of PINK1 or Parkin can recue MPTP induced neurodegeneration [274–276] or ameliorate aSYN mediated toxicity [277, 278].	– Compounds increasing PINK1/Parkin are currently not tested in clinical trials.

Pioglitazone, a peroxisome proliferator-activated receptor gamma (PPARγ) agonist, also has been considerably studied in PD. In animal studies, it reduced neuroinflammation, suppressed nitric oxide synthase activity, improved proteasomal clearance, and enhanced mitochondrial biogenesis [264, 279]. However, a phase II clinical trial in early PD patients found no clinical benefit of 44 weeks treatment with pioglitazone on disease progression [265].

As outlined above, mitochondria are highly dynamic organelles that form a complex network within the cell soma, axon and down to the synaptic buttons. Maintaining this network in a viable state relies on constant spatial redistribution via mitochondrial trafficking, as well as balanced mitochondrial fusion and fission, to keep a pool of healthy mitochondria at any time. However, many of these mitochondrial quality control processes appear to be disrupted in PD patients [67]. Based on that, several preclinical approaches have been developed to correct this putative defect in mitochondrial dynamics (Table 3). Inhibition of mitochondrial fission via the mitochondrial division inhibitor 1 (mdivi-1) has been reported to be neuroprotective in an aSYN overexpression rat model. Treatment with mdivi-1, reduced mitochondrial fragmentation and was simultaneously associated with reduced oxidative stress and improved mitochondrial health [267]. Further, accumulation of the mitochondrial adaptor protein Miro on the outer mitochondrial membrane has been identified in PD and linked to delayed mitophagy in experimental PD models [271]. Pharmacological reduction of Miro in cellular and PD Drosophila fly models was able to restore mitophagy and decrease neuronal cell loss [272].

Further, gene therapy approaches targeting PINK1 and Parkin deficiencies have been explored (Table 3). PINK1 overexpression not only ameliorated mitochondrial dysfunction resulting from prior induced PINK1 deficiency in PINK1 mutant Drosophila models [71, 280], but also was protective in an aSYN induced phenotype in aSYN transgenic Drosophila PD model [277], and protected against neuronal loss and mitochondrial dysfunction in in vitro and in vivo MPTP models [274]. Overexpression of parkin has similar effects [275, 276]. A protein-based therapy using a cell-permeable Parkin was protective in 6-hydroxydopamine (6-OHDA) and adeno-associated viral vector (AAV) mouse models, presumably by enhancing mitochondrial quality control via facilitating mitochondrial biogenesis, and promoting mitophagy [278]. It should be noted however that the predictive validity of both the 6-OHDA and MPTP models of PD is questionable, as all of the failed drugs have passed this test in preclinical work.

### Limiting mitochondrial stimulation as a new therapeutic approach

As outlined above, most of the mitochondrially-targeted, disease-modifying strategies that have moved to clinical trials, or are in the planning stages, are aimed at either limiting the consequences of mitochondrial damage (e.g., CoQ10), enhancing the clearance of damaged mitochondria (e.g., Miro targeting) or blunting the inflammatory consequences of mitochondrial dysfunction (e.g., exenatide) [252, 253]. An alternative strategy is to first diminish mitochondrial damage. The mechanistic studies focusing on the origins of mitochondrial oxidant stress in at-risk neurons (like SNc dopaminergic neurons) point to their feedforward stimulation by plasma membrane L-type Ca^2+^ channels. Inhibiting L-type channels with dihydropyridine negative allosteric modulators lowered mitochondrial oxidant stress and mitophagy in at-risk dopaminergic neurons in animal models [145, 176]. They also diminished mitochondrial oxidant stress in a model of recessive PD [145], and showed neuroprotective effects in the MPTP and 6-OHDA models of PD [281, 282]. More importantly, epidemiological studies have shown that use of dihydropyridines is associated with a reduced risk of developing PD [283, 284]. These observations motivated two clinical trials with the dihydropyridine isradipine. Isradipine was chosen for these trials because it has the highest relative affinity for the sub-class of L-type channel thought to be the most important in driving mitochondrial stress in SNc dopaminergic neurons (channels with a pore-forming Cav1.3 subunit). While initial reports stated that there was no evidence of efficacy in modifying disease progression [285], a subsequent re-analysis reopened the discussion on an extended release formulation of isradipine, suggesting that there may be a disease modifying effect based on the UPDRS assessed progression in patients given 10 mg isradipine per day [286].

### Current gaps in our understanding

Based on our current knowledge of mitochondrial dysfunction in PD, there are at least four major gaps in our understanding.

First, the chain of events arising from mitochondrial dysfunction needs to be more rigorously characterized. As in modern cancer treatment, this would allow combination therapies that maximize biological efficacy and minimize unwanted side-effects of treatment (see Tables 2 and 3).

Second, there need to be more objective, and quantitative measures of disease progression. The reliance upon highly variable clinical rating scales adds an enormous amount of noise to clinical trial outcomes and prevents modest disease-modifying effects to be resolved. These biomarkers should include ones that assess mitochondrial function and dysfunction [253]. Current strategies are mainly focused on improving neuroimaging of cellular bioenergetics (e.g., magnetic resonance spectroscopy). However, studies should also implement blood- or CSF-based biomarkers as recently demonstrated [223].

Third, we need to have a better understanding of the mitochondrial pathways leading to neurodegeneration in the different PD subtypes [287]. This could allow personalized disease-modification therapies and better target engagement.

Fourth, we need to know whether the mechanisms driving disease progression in PD are time invariant or not. It could be that mitochondrial dysfunction is important in the early stages of PD pathogenesis, but not in later stages. For example, the later stages of cell loss in PD could be driven by network dysfunction caused by less than complete disruption of at-risk neuron function. A clear understanding of these mechanisms would allow disease-modifying treatments to be tailored to the respective disease stage.

## Conclusions

Mitochondrial dysfunction is a core hallmark of PD. Preclinical, epidemiological, histopathological, and clinical trial data point towards mitochondrial dysfunction as being a significant disease driving factor in idiopathic and familial PD. On the cellular level, core features are CI impairment, increased oxidative stress, disturbed mitochondrial quality control mechanisms, and bioenergetic deficiency. Current experimental evidence indicates that there are several mitochondrial pathways that contribute to PD pathogenesis. Targeting more than one of these pathways at the same time may be a more effective strategy than trying to affect just one. Moreover, given that the pathology in PD is largely in the brain, drug delivery strategies that optimize brain delivery and target engagement need to be pursued. So, while no treatment has been unequivocally shown to slow disease progression in the early stage of PD, there remains optimism that this situation will change soon.

## Data Availability

N/A.

## Abbreviations

6-OHDA: 6-hydroxydopamine
AAV: Adeno-associated viral vector
aSYN: Alpha-synuclein
ATP: Adenosine triphosphate
ATP13A2: ATPase type 13A2
CI: Mitochondrial complex I
CII: Mitochondrial complex II
CIII: Mitochondrial complex III
CIV: Mitochondrial complex IV
CHCHD2: Coiled-Coil-Helix-Coiled-Coil-Helix Domain Containing protein 2
CoQ10: Coenzyme Q10
DA: Dopamine
DaTSCAN: Dopamine transporter ioflupane imaging
DLP1: Dynamin-1-like protein
DJ1: Protein deglycase DJ1
ER: Endoplasmic reticulum
ETC: Electron transport chain
FBXO7: F-box protein 7
GBA1: β-glucocerebrosidase
GLP-1: Glucagon-like peptide 1
HSP31: Heat shock protein 31
IPSCs: Induced pluripotent stem cells
L-DOPA: Levodopa
LP: Lewy pathology
LRRK2: Leucine-rich repeat kinase 2
mdivi-1: Mitochondrial division inhibitor 1
MPP^+^: 1-methyl-4-phenylpyridinium
MPPP: 1-methyl-4-phenyl-4-propionoxy-piperidine
MPTP: 1-methyl-4-phenyl-1,2,3,6-tetrahydropyridine
mtDNA: Mitochondrial DNA
NAC: N-Acetyl cysteine
NAD: Nicotinamide adenine dinucleotide
OXPHOS: Oxidative phosphorylation
PARK: Familial PD genes
Parkin: E3-Ubiquitin-protein-Ligase Parkin
PD: Parkinson’s disease
PINK1: PTEN-induced kinase-1
PPARγ: Peroxisome proliferator-activated receptor gamma
POLG: Gene coding for DNA polymerase gamma
ROS: Reactive oxygen species
SNc: Substantia nigra pars compacta
SNCA: Gene coding for alpha-synuclein
TOM: Translocase of outer mitochondrial membrane
UDCA: Ursodeoxycholic acid
VPS35: Vacuolar protein sorting 35
VPS13C: Vacuolar protein sorting 13 homolog C
